# Aortic Stenosis: Diagnosis, Molecular Mechanisms and Therapeutic Strategies—A Comprehensive Review

**DOI:** 10.3390/jcm14144949

**Published:** 2025-07-12

**Authors:** Cosmin Marian Banceu, Daiana Cristutiu, Simona Gurzu, Marius Mihai Harpa, Diana Banceu, Horatiu Suciu

**Affiliations:** 1 Department ME 2, George Emil Palade University of Medicine, Pharmacy, Science, and Technology of Targu Mures, 540139 Targu Mures, Romania; cosmin.banceu@umfst.ro (C.M.B.);; 2 Emergency Institute for Cardiovascular Diseases and Transplantation Targu Mures, 540136 Targu Mures, Romania; 3 Department of Pathology, George Emil Palade University of Medicine, Pharmacy, Science, and Technology of Targu Mures, 540139 Targu Mures, Romania; 4 Research Center for Oncopathology and Translational Medicine (CCOMT), George Emil Palade University of Medicine, Pharmacy, Science, and Technology of Targu Mures, 540142 Targu Mures, Romania; 5 Department M 3, George Emil Palade University of Medicine, Pharmacy, Science, and Technology of Targu Mures, 540139 Targu Mures, Romania; 6 Organizing Institution for Doctoral University Studies, West University of Timisoara, 300223 Timisoara, Romania

**Keywords:** aortic stenosis, heart team strategy, TAVR, SAVR, individual risk profiles

## Abstract

Aortic stenosis (AS) is a progressive valvular heart disease marked by a restriction of blood flow through the aortic valve, resulting in considerable morbidity and mortality if not addressed. AS has historically been managed through surgical aortic valve replacement (SAVR), but there is a growing trend towards the use of transcatheter aortic valve replacement (TAVR). TAVR has transformed the management of symptomatic severe AS and is currently authorized for patients with varying levels of surgical risk. The rising application of TAVR in patients under 65 years presents a challenge for heart valve teams (HVTs) managing younger individuals whose life expectancy may surpass the durability of the valve. Patients over 65 years are typically treated with bioprosthetic tissue valves; however, there remains significant uncertainty regarding the selection between TAVR and SAVR.

## 1. Introduction

Aortic valve replacement, either through surgery or interventional treatment, is warranted for survival enhancement, symptom alleviation, and maintenance of left ventricular function in individuals with severe aortic valve disease [1,2]. Aortic stenosis is the predominant valve disease in the Western world, with an estimated prevalence ranging from 0.2% in individuals aged 50 to 59 years to nearly 10% in those aged 80 to 89 years. As noted by Roleder et al., aortic stenosis not only has a high rate of morbidity and mortality but also places a significant financial burden on healthcare systems [3]. Surgical aortic valve replacement (SAVR) has been progressively supplanted with transcatheter aortic valve replacement (TAVR) in the conventional management of aortic stenosis [4]. TAVR has demonstrated noninferiority and even superiority over SAVR for mortality, stroke, and rehospitalization in randomized clinical trials [5,6]. The terms TAVI (transcatheter aortic valve implantation) and TAVR (transcatheter aortic valve replacement) are used interchangeably in the literature, with “TAVR” being more common in North America and “TAVI” preferred in Europe, reflecting regional differences in terminology rather than procedural technique.

**Figure 1:**
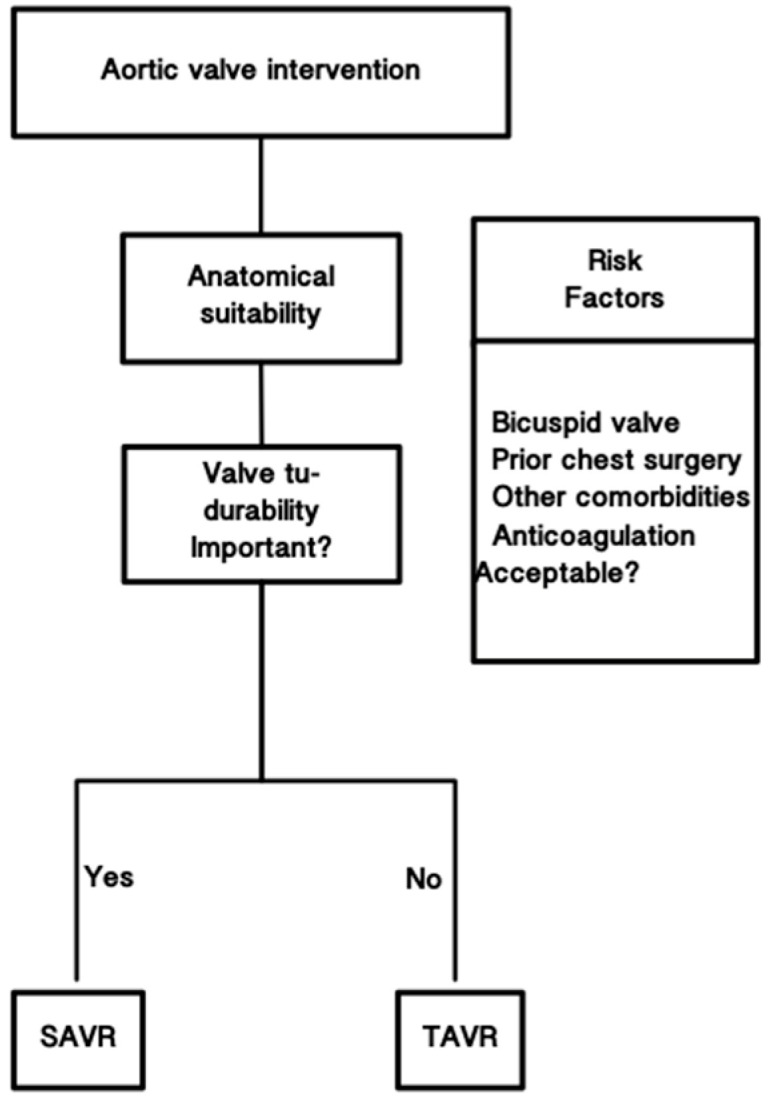
Strategy employing shared decision-making.

TAVR is sanctioned for all risk categories of patients necessitating aortic valve intervention for calcific aortic stenosis and, according to STS/TVT (Society of Thoracic Surgeons/Transcatheter Valve Therapy) data, has become the primary intervention method for these patients [5,6,7,8,9,10]. In cases where transfemoral access is not feasible, alternative vascular approaches for TAVI—including transaxillary, transsubclavian, transcarotid, transcaval, transaortic, and transapical routes—may be considered based on patient-specific anatomical and clinical factors [11,12]. The utilisation of TAVR for aortic stenosis has surged significantly due to the broadened indications based on risk stratification, making TAVR the predominant treatment modality for this patient demographic, irrespective of age [10]. This has been demonstrated in multiple investigations [10,11,12,13,14,15]. This rise in utilisation may be partially ascribed to patient choice for a less invasive method. A benefit-risk study conducted by Marsh et al. indicated that patients under 60 would accept heightened clinical risks linked to TAVR in return for the advantages of its minimally invasive characteristics [16]. The rising utilisation of TAVR in individuals under 65 years has presented a dilemma for heart valve teams (HVTs) managing younger patients whose life expectancy may surpass valve durability. In patients with asymptomatic severe aortic stenosis, the incidence of death, stroke, or unplanned cardiovascular hospitalization was significantly lower with an early transcatheter aortic valve replacement (TAVR) strategy rather than clinical surveillance [17]. A multidisciplinary, patient-centred treatment strategy employing shared decision-making (SDM) that integrates anatomical considerations, evidence-based procedures, and patient preferences are crucial to bridge the disparity between recommended actions and patient aspirations (Figure 1). The correlation between age and risk is ambiguous, as low-risk comparative studies of TAVR and surgical aortic valve replacement (SAVR) involved a limited number of patients under 65 years old [6,7]. In the PARTNER-3 trial, merely 6.9% and 7.9% of participants in the TAVR and SAVR groups were under 65 years of age [6]. Consequently, the findings of the low-risk TAVR versus SAVR trial exhibit restricted applicability to younger patients.

Patients with aortic stenosis must make a critical decision regarding valve selection: bioprosthetic tissue valves, which closely resemble and function like natural valves, possess limited longevity; conversely, mechanical surgical valves, known for their durability but necessitating lifelong anticoagulation, correlate with increased life expectancy in individuals aged 50 to 70 years [18].

The selection between surgical and transcatheter treatment, as well as mechanical and bioprosthetic valves, is contingent upon various parameters such as age, life expectancy, anticoagulation tolerance, and individual preferences. Mechanical valves are typically favoured for younger patients who have a higher life expectancy and a reduced risk of bleeding [19]. Mechanical and bioprosthetic valves are equally endorsed for patients aged 50 to 65 years, according to Heidenreich et al. (2022) [20]. Nonetheless, significant ambiguity exists between SAVR and TAVR for individuals over 65 years, with vague guideline recommendations loosely predicated on age.

Age thresholds serve as proxies for life expectancy, as patients under 65 without substantial comorbidities are anticipated to have a longer lifespan and are thus more prone to encounter long-term post-procedural problems. Patients with severe aortic stenosis treated with surgery or TAVR at 5 years had similar risks of debilitating stroke or all-cause death [21]. This further substantiates the endorsement of SAVR for this demographic [19,22].

## 2. Pathophysiology

Aortic stenosis results from an inflammatory process initiated by endothelial damage from mechanical stress, followed by lipid infiltration, fibrosis, leaflet thickening, and ultimately calcification [23]. Calcific aortic stenosis leads to elevated leaflet stiffness and a constricted aortic valve orifice, resulting in a pressure gradient across the valve [24]. Aortic stenosis features an extended subclinical phase characterized by aortic sclerosis, during which calcification occurs on the valve without the presence of a transvalvular gradient. Advanced constricting of the aortic valve, accompanied by left ventricular pressure overload and subsequent left ventricular hypertrophy, results in the classic triad of symptoms associated with aortic stenosis: heart failure, syncope, and angina [25].

In calcific aortic stenosis, the valve cusps undergo progressive thickening, fibrosis, and calcification. This leads to elevated valve stiffness, diminished cusp excursion, and gradual narrowing of the valve orifice, which differs from the cusp fusion observed in rheumatic heart disease. Calcific aortic stenosis results from active inflammatory processes that involve biochemical, humoral, and genetic factors. The starting event in atherosclerosis is thought to be endothelial damage caused by heightened mechanical stress and diminished shear stress. This leads to a distinct course of lesions in the stenotic valve. Endothelial injury or disruption can facilitate the penetration of lipids into the valvular endothelium, leading to accumulation in inflammatory regions [26,27]. Lipoproteins associated with atherogenesis, such as low-density lipoprotein and lipoprotein(a), are found in early aortic valve lesions [26] and are subject to oxidative modification [28]. Oxidized lipoproteins exhibit significant cytotoxicity and can induce pronounced inflammatory responses and subsequent mineralization [28]. Endothelial damage combined with lipid deposition initiates inflammation in the valve. Inflammatory cells and cytokines play a crucial role in stimulating and establishing the fibrotic and calcific processes that contribute to increased valve stiffness. Haemorrhage associated with these vessels is significant, occurring in 78% of patients with severe AS and linked to neovascularisation, macrophage infiltration, and accelerated disease progression [29,30]. The stenotic aortic valve exhibits significant thickening, resulting from fibrous tissue building up and extracellular matrix remodelling [31]. The renin–angiotensin system is believed to influence this fibrotic process. In stenotic aortic valves, both tissue angiotensin-converting enzyme (ACE) and angiotensin II are up-regulated, with angiotensin receptors identified on valve myofibroblasts [32]. Valve calcification is a significant factor in the progression of aortic stenosis and can be measured through computed tomography. Valvular calcification is associated with valve severity [33], disease progression [34], and the onset of symptoms and adverse events [35]. In the initial phases of aortic stenosis, calcification consists of nodules that contain hydroxyapatite, which is deposited on a bonelike matrix comprised of collagen, osteopontin, and various other bone matrix proteins [36,37,38]. As aortic stenosis progresses, remodelling of the calcification occurs, leading to the identification of lamellar bone, microfractures, and hematopoietic tissue within the valve in the later stages of the disease [37].

**Figure 2:**
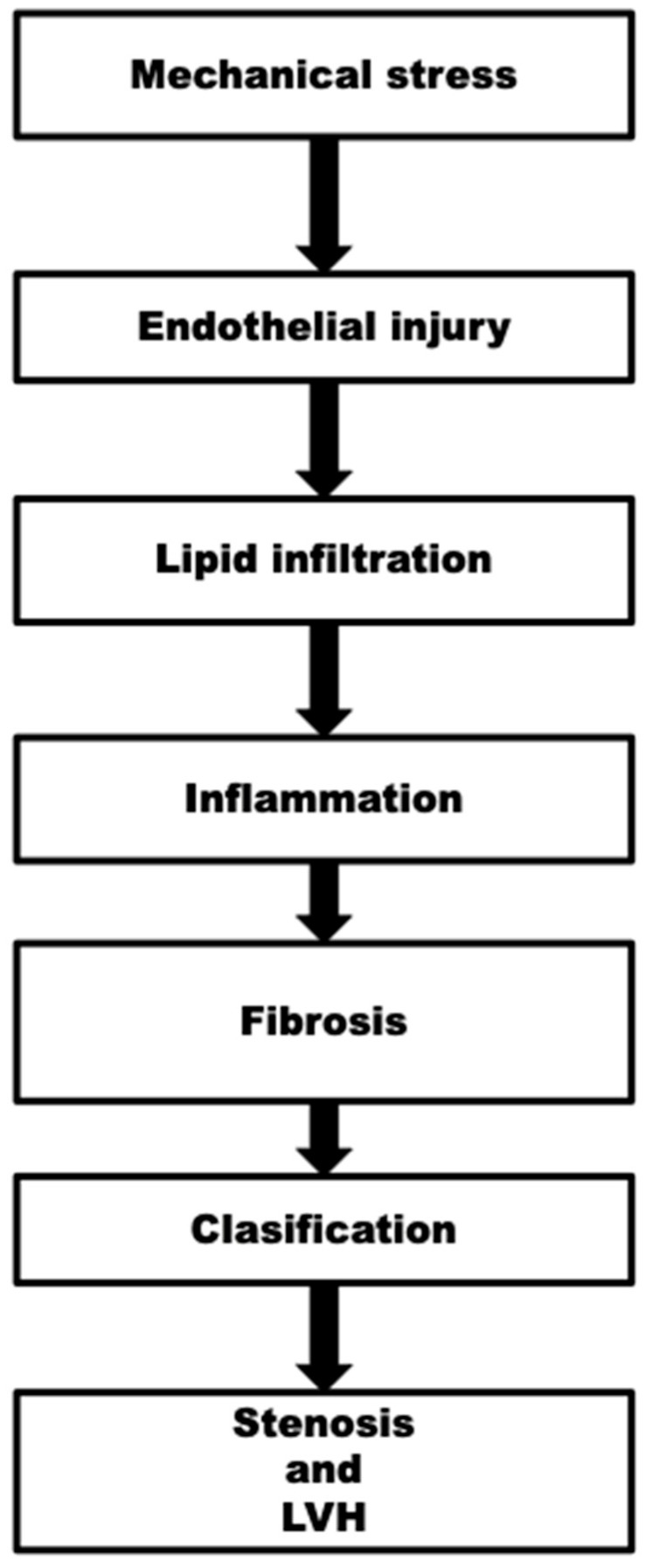
Pathophysiology of aortic stenosis.

Aortic stenosis is defined as the narrowing of the aortic valve opening, resulting in the obstruction of blood flow from the left ventricle to the aorta during systole (Figure 2).

The obstruction elevates pressure in the left ventricle, necessitating increased effort to pump blood through the constricted valve. Chronic elevated pressure leads to left ventricular hypertrophy (LVH), characterized by the thickening of the heart muscle to accommodate the heightened workload. Initially, this hypertrophy aids in sustaining cardiac output in the presence of stenosis. As AS advances, the compensatory mechanisms of the heart may become insufficient, resulting in cardiac dysfunction. The hypertrophied myocardium may exhibit increased stiffness, impairing its capacity to relax and adequately fill during diastole, potentially resulting in heart failure [38]. Aortic stenosis commonly presents with exertional dyspnea, fatigue, angina or syncope. Untreated severe aortic stenosis can result in considerable cardiac complications, such as heart failure and arrhythmias [39].

## 3. Role of Genetics

**Figure 3:**
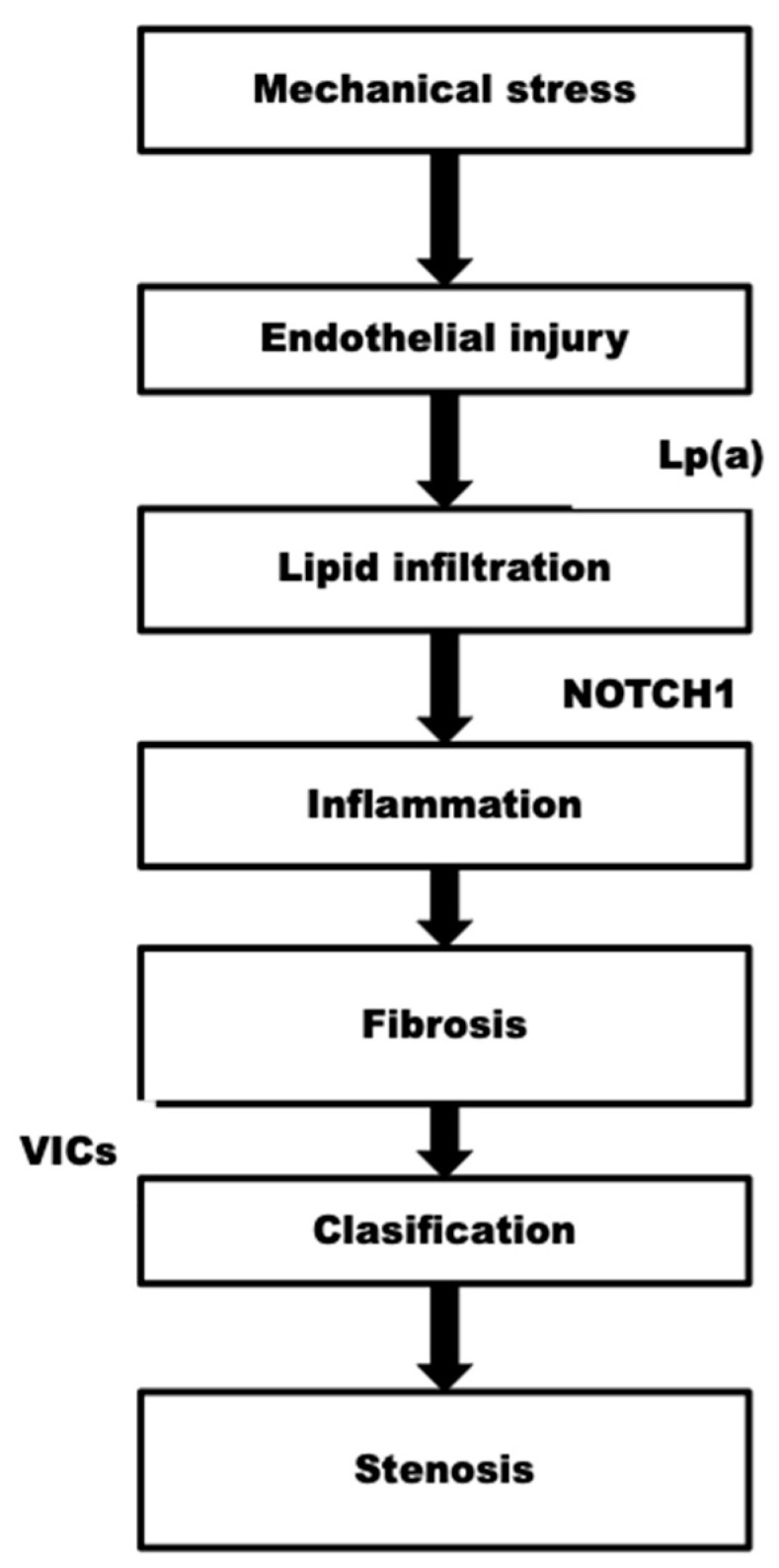
Role of genetics.

The growth phase of calcific aortic stenosis (CAS) is characterized by recurrent fibrosis and calcification [40,41], stimulated valvular interstitial cells (VICs) in inflammation promote fibrosis via the secretion of matrix metalloproteinases, and adopting a myofibroblastic phenotype [41,42]. The scarred tissue serves as a nidus for calcification, where inflammation-induced apoptosis of VICs results in diffuse microcalcification due to the release of apoptotic bodies [43]. Microcalcification is enhanced by releasing calcifying microvesicles from vascular interstitial cells and macrophages [43,44]. Subsequently, VICs induce macrocalcification by adopting an osteoblast-like phenotype, a process facilitated by the dysregulation of osteogenic mediators, including bone morphogenic protein 2 and NOTCH1 [40,41,45,46,47]. Lipoprotein(a) (Lp(a)) was previously demonstrated to promote VIC differentiation [48]. The advancement of AS is multifactorial, with both Lp(a) levels and NOTCH1 function being affected by genetic variance [47,48,49] (Figure 3).

Furthermore, innovative therapies aimed at Lp(a) demonstrate potential based on the molecule’s established structure and its involvement in CAS pathophysiology, along with its associated genetics and function as a measurable biomarker [50,51,52,53,54,55,56].

Increased Lp(a) enhances the development of atherosclerosis associated with CAS, affecting an estimated one billion individuals with high levels [57,58,59]. The recognised polymorphism locus rs1045872 in recessive patients promotes the causal role of Lp(a) concentration in calcification development [50]. Genetics offers a basis for the screening and quantification of circulating Lp(a), which may aid in stratifying at-risk patients for medical intervention [60,61]. The feasibility of genetic screening is underscored by the fact that Lp(a) represents the sole monogenic risk factor for AS [62].

## 4. Contribution of miRNA

A substantial amount of research was conducted to investigate the role of miRNAs in AS. Researchers frequently evaluate miRNAs through microarrays and confirm findings using quantitative reverse transcription–polymerase chain reaction (qRT-PCR). Among those exhibiting high consistency, miR-21 and miR-204 are notable mentions. MiR-21 was observed to be increased in patients with AS. Despite this, miR-21 exhibits significant potential biological functions, making it challenging to ascertain its involvement in AS without additional research [62,63,64,65,66,67]. In contrast, several studies have reported that miR-204 is downregulated [68,69,70,71]. Similar to miR-21, miR-204 has the tendency to target multiple genes, complicating the establishment of cause-and-effect relationships. A significant limitation exists, namely, the absence of more recent analyses. Conversely, miR-22 and miR-133 deserve investigation due to certain inconsistencies. Yeang et al. recently found that miR-22 has been decreased in the valves of patients with calcific aortic valve disease (CAVD) [62]. Coffey et al. presented results that were partly distinct but appropriately explicable a few years prior: patients with AS without concurrent CAD exhibited reduced levels of miR-22, consistent with the findings of Yeang et al. [62,64]. Patients with AS and concurrent CAD exhibited elevated levels of miR-22 in comparison to controls. Research indicates that miR-133 is downregulated in aortic stenosis [72,73]. Higher levels of miR-133 were repeatedly identified as an independent predictor of left ventricular mass reduction one year post-AVR. The findings from the study by Fabiani et al. concerning miR-133a are particularly noteworthy [67]. Fabiani et al. observed that miR-133 is upregulated in patients with aortic stenosis and reduced ejection fraction, a finding that was unexpected [67]. MicroRNAs may serve as predictors for the outcomes of interventional treatments, specifically aortic valve replacement (AVR) or transcatheter aortic valve replacement (TAVR) [67]. In this context, they would be crucial, particularly for patients on the threshold of eligibility for AVR or TAVR.

## 5. Immunohistochemistry Aspects

Immunohistochemical studies have yielded significant findings in the pathophysiology of aortic stenosis (AS), especially regarding the cellular and molecular mechanisms that contribute to the disease’s progression. Studies demonstrate that CAVD exhibits characteristics akin to atherosclerosis, indicating a potential inflammatory origin. Immunohistochemical analyses of CAD have revealed the presence of various cellular infiltrates, such as T lymphocytes (CD3+), B lymphocytes (CD20+), and macrophages (CD68+). The findings indicate the role of immune cells in the inflammatory process of the valves [74]. Endothelial cells are essential for the maintenance of valvular function. CD31 (PECAM-1) is frequently utilised as a marker for the identification of endothelial cells in tissue sections. Modifications in endothelial cell markers in the context of AS may indicate endothelial dysfunction, which contributes to disease progression [75]. Oxidative stress plays a significant role in valvular calcification. Immunohistochemical studies indicate that oxidative stress markers are increased in valvular endothelial cells during the advancement of CAVD, suggesting their role in endothelial dysfunction and subsequent valvular calcification [76]. Immunohistochemistry has proven essential in clarifying the inflammatory, endothelial, and molecular changes linked to aortic stenosis, enhancing the understanding of its pathogenesis and identifying potential therapeutic targets.

## 6. TAVR vs. SAVR

### 6.1. Anatomy

Anatomic features that elevate the risk of SAVR, such as previous coronary bypass, hostile chest, or past chest radiation, can affect surgical candidacy. The existence of a calcified or porcelain aorta elevates the surgical potential for myocardial infarction, stroke, and haemorrhage; hence, these patients were excluded from the low-risk trials comparing SAVR to TAVR [5,6,77]. Preprocedural preparation might anticipate risk and adjust the approach by opting for a smaller valve or decreasing the inflation capacity as opposed to picking a self-expanding valve. Multidetector computed tomography (MDCT) is optimal for operational scheduling in patients with bicuspid aortic stenosis (AS) undergoing either surgical aortic valve replacement (SAVR) or transcatheter aortic valve replacement (TAVR). In relation to SAVR, the preoperative evaluation includes the assessment of the location and height of the coronary arteries to guarantee adequate distance to the coronary ostia and sufficient room in the sinuses for potential valve-in-valve TAVR upon failure of the initial valve. The surgeon must consider the height of the stitching cuff, as it will reduce the distance to the coronary. In TAVR, patients with bicuspid aortic stenosis frequently necessitate valve resizing to accommodate the elliptical orifice and calcification. The evaluation of these anatomical trade-offs is most effectively conducted with MDCT. Excessive calcification of the leaflet and calcified raphe predicts operational difficulties and mid-term mortality in TAVR [78]. The likelihood of coronary occlusion with native valve TAVR is minimal; however, the death rate is 50% [79]. A meticulous evaluation of pre-TAVR MDCT is essential for assessing the risk of coronary obstruction, focusing on coronary height, leaflet length, calcium volume and distribution in the leaflets, and the distance between the valve and coronary arteries. It is imperative that structural cardiologists and cardiac surgeons managing AS possess expertise in MDCT preoperative measures and independently evaluate anatomical risks without succumbing to industry influence.

In patients with bicuspid aortic stenosis requiring surgical aortic valve replacement, replacement of the ascending aorta and/or sinuses/root is warranted when the diameter reaches or exceeds 4.5 cm [19,80]. Patients undergoing isolated SAVR or TAVR with a 4.0- to 4.5 cm ascending aorta necessitate lifelong monitoring [80]. They may also need aortic surgery at later age due to increasing ascending aortopathy [81]. Evidence indicating the generally advantageous surgical risk profile of TAVR compared to SAVR exists [82], although information about the long-term consequences of the procedure is few [83], particularly within the <70 age demographic. Evidence indicates that redo SAVR yields superior haemodynamic outcomes compared to ViV (valve-in-valve) TAVR, particularly in patients with tiny surgical valves [84]. Prosthesis–patient mismatch (PPM) arises when a prosthetic valve possesses a diminished indexed effective orifice area, resulting in adverse haemodynamics. It is linked to heart failure, readmission, and elevated mortality rates. Prosthesis–patient mismatch poses significant challenges in younger patients and individuals with left ventricular failure [85]. The Global Valve-in-Valve International Data (VIVID) registry indicates that around 30% of patients received a prosthesis measuring 21 mm or smaller in the aortic position [86], resulting in a 31.8% prevalence of PPM following valve-in-valve (ViV) procedures. Conduction disturbances represent common complications following TAVR, with pacemaker implantation rates ranging between 6.5 and 25.9%, particularly with self-expanding valves, compared to lower rates observed after SAVR (3–5%) [87,88]. New-generation surgical prostheses, including those incorporating novel tissue bioprosthesis and frames designed to accommodate future TAVR procedures, offer promising options for younger patients or those at increased risk of reintervention [89]. A recent meta-analysis indicated that ViV TAVR is linked to a hazard ratio of 4.63 for significant prosthesis–patient mismatch in comparison to redo SAVR [90]. TAVR may be inapplicable for a significant subset of patients, as optimal ViV outcomes are contingent upon patient anatomy and valve compatibility [91]. Data comparing redo surgery AVR and ViV TAVR is limited, with current evidence indicating no superiority of ViV TAVR over redo surgery AVR for mid-term mortality [92]. Regarding bioprosthetic valve’s inclination to eschew anticoagulation, studies indicate that 20% of patients after AVR experience atrial fibrillation during mid- and long-term follow-up [93]. A substantial number of patients receiving valve replacement necessitate anticoagulation irrespective of the valve type.

### 6.2. Concomitant Disease

Guidelines advise SAVR and coronary artery bypass grafting for AS patients with complicated left main bifurcation disease or multivessel disease with a SYNTAX score higher than 33, rather than TAVR and percutaneous coronary intervention [19]. Diabetes drastically alters risk profiles and shared decision-making in diabetic patients, and its effects on stenting outcomes are more negative than those of bypass surgery [94]. Surgery is advised for patients with low- and intermediate-risk primary mitral regurgitation, with combined SAVR and mitral repair or replacement as the standard technique [19]. Secondary mitral regurgitation ameliorates following successful aortic valve replacement in roughly 50% of instances, indicating that certain patients may not necessitate mitral valve repair [95,96].

### 6.3. Valve Durability

All bioprosthetic valves (Bv) undergo deterioration and malfunction gradually. The criterion for the long-lasting effectiveness of surgical valves is survival with no need for reintervention. Employing freedom from reoperation as a metric for assessing structural valve degeneration underrepresents its actual prevalence. There is a deficiency of long-term clinical trial data comparing structural valve dysfunction (SVD) after SAVR and TAVR. Previous studies included high-risk and elderly patients with restricted life expectancy, and various enhancements in TAVR design have complicated comparisons.

Thyregod et al. documented reduced 10-year incidences of severe SVD (1.5% vs. 10.0%) and non-SVD (10.2% vs. 31.9%) with CoreValve TAVR compared to SAVR, attributed to diminished severe PPM and mean gradients, although they observed no disparities in the rates of clinical valve thrombosis, endocarditis, or bioprosthetic valve failure [95]. Mack et al. revealed that the 5-year severe structural valve deterioration (SVD) rates were the same for both Sapien 3 and surgical aortic valve replacement (SAVR), as were the rates of SVD-related valve failure and reintervention [8]. Forrest et al. indicated that the four-year reintervention rates were minimal, at 1.3% for TAVR compared to 1.7% for SAVR [9]. O’Hair et al. presented a 5-year pooled analysis of bioprosthetic valve malfunction rates, indicating rates of 15.4% for SAVR compared to 9.6% for TAVR. Independent of AVR therapy, bioprosthetic valve malfunction correlated with a five-year heightened risk of mortality, cardiovascular mortality, and rehospitalisation [97].

### 6.4. Mechanical vs. Bioprosthetic Aortic Valve Replacement

Selecting a surgical valve for middle-aged individuals necessitates that the surgeon and patient evaluate the risks and advantages of each valve type while considering patient preferences and lifestyle factors [98]. Mechanical valves are linked to prolonged durability and a low incidence of reintervention; nevertheless, they are also related with thrombogenicity, the necessity for lifetime anticoagulation, and the potential for bleeding problems, each of which can significantly affect quality of life [99,100]. Bioprosthetic valves do not necessitate lifelong anticoagulation and present significantly reduced risks of thromboembolism and haemorrhage; however, structural valve deterioration (SVD) may develop over time, requiring reintervention [101,102,103,104,105,106]. In younger patients, SVD appears to be expedited [100,107,108,109]. The latest developments in percutaneous bioprosthetic valves introduce an additional aspect to a method aimed at circumventing both anticoagulation and repeat sternotomy [108,109]. The utilisation of mechanical valves (MVs) for surgical aortic valve replacement (SAVR) has significantly diminished over the last two decades.

The primary trade-off is the risk of reoperation with biological valves vs. the difficulties linked to long-term anticoagulation with mechanical valves [110]. Randomised clinical data comparing contemporary prostheses are scarce [111,112], although a well-documented trend indicates a rising preference for biological valves over mechanical valves in recent decades, in both aortic and mitral positions [113]. The utilisation of biological valves in patients under 70 years of age has risen in recent years for both aortic and mitral locations [113,114]. Despite being primarily influenced by evolving recommendations that permit a broader age range for both prostheses, there has been no significant alteration in the existing evidence [115,116]. Previously, mechanical valves were predominantly deemed suitable for younger patients because to their superior longevity; however, advancements in the durability of bioprosthetic valves have resulted in their increased utilisation [110]. Conflicting evidence regarding middle-aged patients complicates the decision between mechanical and bioprosthetic valves [110,117,118,119]. The survival rate ranged from 89% to 97% among patients aged 65 years or younger [119,120,121,122]. The reasons for the discrepancies in survival rates among these studies remain ambiguous, as the studies with contradictory data encompassed patients with older-generation valves [123,124] and those with newer-generation valves [125,126].

**Figure 4:**
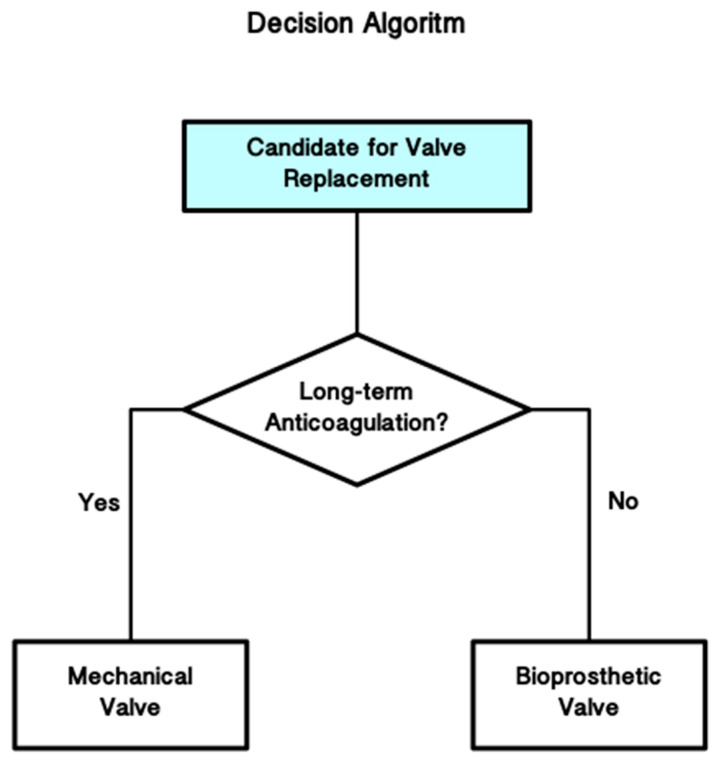
Decision algoritm.

The lack of a notable survival advantage linked to one type of prosthesis over another directs decision-making towards lifestyle factors, such as the burden of anticoagulation therapy and monitoring, as well as the comparative risks of major morbidity, chiefly stroke, reoperation, and significant bleeding incidents (Figure 4).

Adverse effects related to valves in younger patients were similar across published trials. The five-year rates of independence from reintervention for middle-aged patients vary from 91.5% to 96.3% [119,120,121,122]. The five-year rates of independence from thromboembolic events range from 95% to 98.1% [121,122]. The five-year independence from endocarditis rates are between 97% and 95.8% in middle-aged individuals [119,122].

Prior meta-analyses addressing this matter yielded incongruent findings [127,128], although these studies excluded the research conducted by Goldstone et al., which is the most extensive observational study comparing the outcomes of MVs against BVs, categorised by age [109]. Zhao et al. determined that MVs and BVs present comparable mortality risks [126]. Regrettably, the conclusion was constrained as around fifty percent of the research included were observational trials that did not employ propensity score matching (PSM) or inverse probability weighting (IPW). The overall mortality estimate for BVs was elevated, and the meta-analysis conclusion was derived from a subgroup analysis restricted to patients aged 60–70, with 88% of the data originating from observational studies that did not employ propensity score matching (PSM) or inverse probability weighting (IPW). Diaz et al., who exclusively analyzed RCTs and PSM studies, identified a stronger correlation between BVs and mortality [127]. Published observational studies employing propensity score matching (PSM) or inverse probability weighting (IPW) methodologies are constrained by restricted sample sizes. The alteration coincided with a transformation in clinical practice, leading to the majority of patients aged 50–69 now receiving BVs [113,114,129]. The rationale for this transition requires elucidation. One reason is the clinical evidence released during this period; wherein numerous observational studies predominantly indicated no disparity in mortality between valve types in the 50–69 age demographic [127]. The observed patient preference for BV may be ascribed to the duration of problems. While bleeding, the primary consequence of mechanical valves, is evident immediately post-surgery and thus impacts lifestyle right away, valve reoperation, the principal complication linked to biological valves, is a prospective issue, creating a false sense of security. This bias is extensively documented in the literature [130].

A contributing element to the transition towards BVs is the growing utilisation of transcatheter aortic valve replacement (TAVR) and valve-in-valve (ViV) TAVR procedures. Data indicated that while the total number of surgical aortic valve replacement (AVR) surgeries remained unchanged following the introduction of transcatheter aortic valve replacement (TAVR), it significantly influenced the selection of valves, leading to an 8-year reduction (from 68 to 60) in the age criterion for favouring biological valves (BVs) over mechanical valves (MVs) [131]. This impact arises from two factors: the necessity to “compete” for patients with a less invasive BV surgery, which is typically favoured by patients, and the potential to circumvent the most feared problems associated with BV valve reoperation through the utilisation of ViV TAVR [132].

Future advancements in clinical practice may influence the risk/benefit ratio of BVs compared to MVs. Complications of anticoagulation are influenced by fluctuations in the international normalised ratio (INR). Enhanced management of INR, attainable by home monitoring, diminishes clinical incidents associated with anticoagulation [133]. Reducing the target INR for low-risk patients has demonstrated a decrease in adverse anticoagulation events in individuals with MV [134]. Moreover, the novel MV prosthesis design facilitates a reduced target INR, which has demonstrated a decrease in the incidence of bleeding episodes [135].

Enhancing the outcomes of valve reoperations may result in improved survival rates for BV patients necessitating reoperation. Mortality rates for redo AVR can be as low as 2.5% at proficient centres [110]. Conversely, this is not applicable when analysing extensive databases, where the mortality rate following redo AVR is approximately 9% [110].

Recent guidelines have reduced the suggested age for the usage of mechanical valves (MVs) to patients under 50 years in the American College of Cardiology/American Heart Association guidelines [19], and to under 60 years in the aortic position and 65 years in the mitral position in the European Society of Cardiology (ESC)/European Association for Cardio-Thoracic Surgery (EACTS) guidelines, indicating a likely continuation of this trend [115].

## 7. Conclusions

Aortic stenosis constitutes a notable clinical challenge, especially among ageing populations where its prevalence is increasing. The selection between TAVR and SAVR in patients with AS remains a topic of considerable debate. The integration of available data with patient preferences regarding percutaneous therapies and expedited recovery may lead to the consideration of TAVR as the primary treatment for patients with severe AS. Data regarding long-term survival and prosthetic durability for low-risk TAVR patients under 65 years of age are insufficient. The debates should focus on optimising results for the initial procedure and assessing the likelihood of future reinterventions. Effective patient selection, collaborative decision-making, and a multidisciplinary heart team strategy are crucial for customising therapy according to individual risk profiles and anatomical factors. Next-generation valve designs and minimally invasive procedures are anticipated to enhance treatment strategies. Ongoing research, long-term outcome data, and the refinement of clinical guidelines are essential for ensuring evidence-based, patient-centred care for individuals with AS.

## Data Availability

The data supporting this study’s findings are available from the corresponding author upon request.
